# Latent class evaluation of the performance of serological tests for exposure to *Brucella* spp. in cattle, sheep, and goats in Tanzania

**DOI:** 10.1371/journal.pntd.0009630

**Published:** 2021-08-24

**Authors:** Rebecca F. Bodenham, Stella Mazeri, Sarah Cleaveland, John A. Crump, Folorunso O. Fasina, William A. de Glanville, Daniel T. Haydon, Rudovick R. Kazwala, Tito J. Kibona, Venance P. Maro, Michael J. Maze, Blandina T. Mmbaga, Niwael J. Mtui-Malamsha, Gabriel M. Shirima, Emanuel S. Swai, Kate M. Thomas, Barend M. deC. Bronsvoort, Jo E. B. Halliday

**Affiliations:** 1 Institute of Biodiversity, Animal Health and Comparative Medicine, College of Medical Veterinary and Life Sciences, University of Glasgow, Glasgow, United Kingdom; 2 The Epidemiology, Economics and Risk Assessment (EERA) group, The Roslin Institute and The Royal (Dick) School of Veterinary Studies, University of Edinburgh, Edinburgh, United Kingdom; 3 Duke Global Health Institute, Duke University, Durham, North Carolina, United States of America; 4 Kilimanjaro Christian Medical Centre, Moshi, Tanzania; 5 Kilimanjaro Clinical Research Institute, Moshi, Tanzania; 6 Centre for International Health, University of Otago, Dunedin, New Zealand; 7 Kilimanjaro Christian Medical University College, Moshi, Tanzania; 8 Division of Infectious Diseases and International Health, Duke University Medical Center, Durham, North Carolina, United States of America; 9 Emergency Centre for Transboundary Animal Diseases, Food and Agriculture Organization (FAO) of the United Nations, Dar es Salaam, Tanzania; 10 Department of Veterinary Tropical Diseases, Faculty of Veterinary Science, University of Pretoria, Onderstepoort, South Africa; 11 Sokoine University of Agriculture, Morogoro, Tanzania; 12 Nelson Mandela African Institution for Science and Technology, Arusha, Tanzania; 13 Department of Medicine, University of Otago, Christchurch, New Zealand; 14 Directorate of Veterinary Services, Ministry of Livestock and Fisheries, Dodoma, Tanzania; Institute of Tropical Medicine, BELGIUM

## Abstract

**Background:**

Brucellosis is a neglected zoonosis endemic in many countries, including regions of sub-Saharan Africa. Evaluated diagnostic tools for the detection of exposure to *Brucella* spp. are important for disease surveillance and guiding prevention and control activities.

**Methods and findings:**

Bayesian latent class analysis was used to evaluate performance of the Rose Bengal plate test (RBT) and a competitive ELISA (cELISA) in detecting *Brucella* spp. exposure at the individual animal-level for cattle, sheep, and goats in Tanzania. Median posterior estimates of RBT sensitivity were: 0.779 (95% Bayesian credibility interval (BCI): 0.570–0.894), 0.893 (0.636–0.989), and 0.807 (0.575–0.966), and for cELISA were: 0.623 (0.443–0.790), 0.409 (0.241–0.644), and 0.561 (0.376–0.713), for cattle, sheep, and goats, respectively. Sensitivity BCIs were wide, with the widest for cELISA in sheep. RBT and cELISA median posterior estimates of specificity were high across species models: RBT ranged between 0.989 (0.980–0.998) and 0.995 (0.985–0.999), and cELISA between 0.984 (0.974–0.995) and 0.996 (0.988–1). Each species model generated seroprevalence estimates for two livestock subpopulations, pastoralist and non-pastoralist. Pastoralist seroprevalence estimates were: 0.063 (0.045–0.090), 0.033 (0.018–0.049), and 0.051 (0.034–0.076), for cattle, sheep, and goats, respectively. Non-pastoralist seroprevalence estimates were below 0.01 for all species models. Series and parallel diagnostic approaches were evaluated. Parallel outperformed a series approach. Median posterior estimates for parallel testing were ≥0.920 (0.760–0.986) for sensitivity and ≥0.973 (0.955–0.992) for specificity, for all species models.

**Conclusions:**

Our findings indicate that *Brucella* spp. surveillance in Tanzania using RBT and cELISA in parallel at the animal-level would give high test performance. There is a need to evaluate strategies for implementing parallel testing at the herd- and flock-level. Our findings can assist in generating robust *Brucella* spp. exposure estimates for livestock in Tanzania and wider sub-Saharan Africa. The adoption of locally evaluated robust diagnostic tests in setting-specific surveillance is an important step towards brucellosis prevention and control.

## Author summary

Brucellosis is a zoonotic disease of global importance. The performance of diagnostic tests is variable in different epidemiological settings. Therefore, estimating the setting-specific performance of diagnostic tests for *Brucella* spp. exposure is important in generating robust surveillance data. The performance of the Rose Bengal plate test (RBT) and a competitive ELISA (cELISA) in detecting *Brucella* spp. exposure at the individual animal-level for cattle, sheep, and goats from Tanzania was estimated. The RBT and cELISA median estimates for specificity were consistently high, whereas median estimates for sensitivity were more variable between tests and across livestock species. A parallel diagnostic testing approach (i.e., testing all animals by both RBT and cELISA, where a sample positive by either test was considered a parallel positive) gave the best overall test performance, as compared to a series approach (i.e., testing all animals by RBT, with RBT positive animals confirmed by cELISA, where a sample positive for both RBT and cELISA was considered a series positive). There is a need to further investigate strategies for implementing parallel testing at the herd- and flock-level. Our findings can assist in generating robust estimates for *Brucella* spp. exposure for livestock in Tanzania and similar settings in sub-Saharan Africa. The adoption of locally evaluated robust diagnostic tests in surveillance is an important step towards the prevention and control of brucellosis.

## Introduction

Brucellosis is a zoonotic disease of global distribution [1]. In humans, brucellosis causes febrile illness with non-differentiating clinical signs, and the disease is often misdiagnosed and mistreated [2]. Direct contacts with animal hosts, and their products, are a main source of human infection [3]. The causative agents of brucellosis are bacteria of the genus *Brucella* [4]. *Brucella melitensis* and *B*. *abortus* are two of the *Brucella* spp. that cause the majority of human infections [5]. The important animal hosts for *B*. *melitensis* and *B*. *abortus* are sheep and goats, and cattle, respectively [6]. However, *B*. *melitensis* may also infect cattle and *B*. *abortus* infection has been reported in sheep and goats [7]. Other *Brucella* spp. infecting livestock species include *B*. *suis* and *B*. *ovis*, largely found in pigs and sheep, respectively [8].

Brucellosis is endemic in many pastoral areas of sub-Saharan Africa [7], where people commonly live in close contact with livestock [9]. Some of the factors that influence the prevalence of brucellosis in livestock kept in pastoral settings include, multi-species herd or flock composition, and large herd or flock sizes [9]. Pastoral settings are considered to have the greatest burden of brucellosis [10], although robust epidemiological data on brucellosis are often lacking in these settings [9].

The control of brucellosis in animal host species is a key approach in the prevention and control of human brucellosis [11,12]. The ability to accurately identify animal host species is important in informing the control of livestock brucellosis and therefore in reducing transmission to humans [13]. Accurate diagnostic tests are important tools in disease surveillance. The evaluation of a diagnostic test is by assessment of a test’s sensitivity and specificity, which pertain to the capacity of a test in indicating the true disease, or exposure, status [14].

There are a wide range of diagnostic tests available for brucellosis, including direct methods such as bacteriological culture or DNA detection, and indirect methods such as serological tests [8]. Isolation of *Brucella* spp. by culture enables confirmation of positive infection status [15]. Although culture specificity is 100% [16], sensitivity can be low. Multiple factors impact on culture sensitivity including: the type of sample collected, the timing of sample collection, the culture media used, and the fastidious nature of *Brucella* spp. which may be easily out-competed *in vitro* by contaminating bacteria [3,16–18]. On top of test sensitivity limitations, the high cost of diagnosis by culture, as well as the need for Biosafety Level 3 laboratory facilities and bacteriological expertise, make culture largely inaccessible in low- and middle-income countries [16].

Serology is the most common diagnostic testing approach for brucellosis [2]. A range of serological tests are available, including: primary binding assays, precipitation tests, agglutination tests, and complement fixation tests [19]. A full list of OIE-approved serological tests can be found in the OIE Manual of Diagnostic Tests and Vaccines for Terrestrial Animals [20]. Serological tests for animal brucellosis are applied to serum to identify an antibody response associated with *Brucella* spp. exposure [16]. Serology is easier and safer to implement than culture [21], making it a more feasible routine diagnostic technique in low- and middle-income countries. However, serological tests differ from culture in that they detect exposure to *Brucella* spp., and do not differentiate between recent and historic infection [8]. Additionally, serological testing for brucellosis can be affected by cross-reactivity with other bacteria that possess similar cell structure, such as *Yersinia enterocolitica* O:9, resulting in false positive reactions [22]. Most serological tests are also unable to differentiate between vaccine-induced *Brucella* spp. exposure (such as *B*. *abortus* S19 and *B*. *melitensis* Rev 1 vaccines) and natural *Brucella* spp. exposure [6].

The performance of serological tests vary and there is no single test suitable for all animal species and all epidemiological scenarios [20]. To improve diagnostic performance, serological testing can be implemented using a screening test in conjunction with a confirmatory or complementary test [20]. Here, we focus on the Rose Bengal plate test (RBT) and the Animal and Plant Health Agency (APHA), UK competitive enzyme-linked immunosorbent assay (cELISA). In Tanzania, there is currently no national standardised and evaluated testing procedure for *Brucella* spp. exposure in livestock [23]. However, the RBT and ELISA (ELISA kit not specified) diagnostic tests are included in the government guidelines for the surveillance of animal brucellosis in Tanzania [24]. The RBT is a rapid, low cost test that requires only basic equipment, lending it to low-resource contexts [16]. ELISA diagnostic tests do require more advanced laboratory infrastructure than the RBT, however, the facilities to implement this diagnostic technique are becoming more common in sub-Saharan Africa [16].

The evaluation of diagnostic tests in the epidemiological setting in which they are implemented is important in understanding setting-specific test performance, especially in the absence of a gold standard approach. The Hui and Walter latent class model can be used to generate estimates of diagnostic test sensitivity, specificity, and disease prevalence where no gold standard is available [25]. The Hui-Walter model requires data for two diagnostic tests from two or more subpopulations [26]. Bayesian adaptation of the Hui-Walter model allows the inclusion of prior knowledge of test sensitivity and specificity, and disease prevalence, based on available scientific information [27].

The performance of the RBT and the APHA cELISA for detection of exposure to *Brucella* spp. in cattle at the individual animal-level has been evaluated previously in African populations. This includes studies that have evaluated the performance of the RBT or this cELISA relative to: known positive animals (defined by positive culture) and known negative animals (*Brucella* free animals); or by latent class analysis [13,17,28–33]. A single study has assessed both the RBT and this cELISA [30]. In this Etman *et al*. study, the Hui-Walter latent class model was used to estimate the sensitivity and specificity of the RBT and the cELISA in the detection of exposure to *Brucella* spp. in cattle. Sensitivity and specificity estimates for the RBT were 0.961 and 0.993, respectively, and for the cELISA were 0.971 and 1, respectively [30]. Studies to evaluate test performance of the RBT or the APHA cELISA in Africa for sheep and goats have not been published. There are examples of similar studies comparing the performance of the RBT or this cELISA by testing with known positive animals (defined by positive culture, a combination of positive serology or PCR and culture, vaccinated, experimentally infected or suspect exposed animals) and known negative animals (*Brucella* free animals) from Europe and the Americas [34–42]. Of these varied studies, RBT sensitivity to *Brucella* spp. exposure has been estimated as low as 0.866 for sheep and goats [38], and specificity as low as 0.327 in goats [39]. For this cELISA, the lowest cELISA sensitivity and specificity estimates for *Brucella* spp. exposure in sheep and goats were 0.885 and 0.985, respectively [36].

In Tanzania, approximately 40% of the population practice traditional, exclusive pastoralism [43]. Both *B*. *melitensis* and *B*. *abortus* have been isolated from people living in a pastoralist community of northern Tanzania [44]. The evaluation of diagnostic test performance, and the development of an evidence-based diagnostic testing strategy for *Brucella* spp. exposure in cattle, sheep, and goats in this context, is thus important. The implementation of a standardised testing procedure for *Brucella* spp. exposure in livestock that incorporates test performance data for multiple animal host species, can assist in generating more robust surveillance data for livestock in Tanzania and similar settings in wider sub-Saharan Africa. Such data can be used to inform and evaluate evidence-based prevention and control activities.

Our study aimed to use Bayesian latent class analysis of cattle, sheep, and goat data from Tanzania to: estimate the sensitivity and specificity of the RBT and this cELISA test at the individual cattle-, sheep-, and goat-level; estimate the seroprevalence of *Brucella* spp. exposure in two subpopulations, pastoralist and non-pastoralist; and estimate the sensitivity and specificity of series and parallel diagnostic testing approaches, using the RBT and cELISA.

## Methods

### Research clearance and ethics

Collection of data used for this study was approved by the Tanzania Commission for Science and Technology, the Tanzanian Ministry of Livestock and Fisheries and by government authorities in the study areas. The Kilimanjaro Christian Medical Centre Ethics Committee, Tanzania (KCMC/535; KCMC/832), the National Institute of Medical Research, Tanzania (NIMR/HQ/R.8a/Vol. IX/1522; NIMR/HQ/R.8a/Vol. IX/2028), the University of Glasgow College of Medical, Veterinary and Life Sciences Ethics Committee, UK, and the Institutional Review Board for Clinical Investigations of Duke University Health System, USA (Pro00037356) gave ethical approval for components of this study. All research conducted was in accordance with the guidelines and regulations of the aforementioned organisations and all participating livestock keepers provided written informed consent.

### Study area

Data collection was conducted in the Arusha, Manyara, and Kilimanjaro Regions of northern Tanzania. Across these neighboring regions there are a mix of livestock keeping production systems including pastoral and agro-pastoral systems [45]. Pastoral production systems typically rely on livestock keeping as the main livelihood activity, whereas agro-pastoral production systems are those in which livestock keeping and crop agriculture both make large contributions to livelihoods [45,46]. Tanzanian national administrative regions are divided into districts. Each district is split into wards, with a number of villages found within each ward. Villages may be further divided into sub-villages.

### Study design

The data for these analyses came from two cross-sectional studies. The first was a bacterial zoonoses study (hereafter referred to as the BacZoo study) conducted in Arusha and Kilimanjaro Regions between September 2013 to March 2015. The second was a social, economic and environmental drivers of zoonoses study (hereafter referred to as the SEEDZ study) implemented in Arusha and Manyara Regions from January to December 2016.

#### BacZoo study

For full study methodology see S1 Appendix. Briefly, a survey of livestock owning households was conducted across six districts: Hai, Longido, Monduli, Moshi Municipal, Moshi Rural and Rombo. A list of the wards within each district was obtained from national census records [47]. Wards were first identified as rural or urban based on the census data. In consultation with district-level government representatives, wards were prospectively classified as one of three agro-ecological settings: peri-urban, agro-pastoral or pastoral. Urban wards within Hai, Moshi Municipal, Moshi Rural and Rombo Districts were classified as peri-urban. Rural wards within Hai, Moshi Municipal, Moshi Rural and Rombo Districts that did not contain a majority population of pastoralist livestock keepers were classified as agro-pastoral. Rural wards within Longido and Monduli Districts that contained a majority population of pastoralist livestock keepers were classified as pastoral.

A multistage sampling approach was adopted to select wards, villages, sub-villages and livestock owning households for inclusion in the study. Six wards were selected at random from each agro-ecological setting to give a total of 18 study wards. One village or sub-village (depending on the smallest unit applicable) from each ward was randomly selected for inclusion. Households were randomly selected from a list of livestock owning households generated through consultation with local community leaders in each location. A minimum of five households were selected in each village or sub-village. At each household, up to 15 cattle, sheep, and goats were selected for sampling.

#### SEEDZ study

Full study methodology has been described elsewhere [45,46]. Briefly, a survey of livestock keeping households was conducted in nine districts: Longido, Monduli, Arusha, Karatu, Meru, Ngorongoro, Babati Rural, Mbulu, and Simanjiro. Village lists were obtained from national census data [47]. Villages in wards specified in the census data as urban, were excluded from the selection procedure for this study. Villages within the Ngorongoro Conservation Area were also excluded. In consultation with district-level government representatives, the remaining rural villages were classified as one of two agro-ecological settings: pastoral (where livestock rearing was considered to be the primary livelihood activity), or mixed (where a combination of livestock keeping and crop agriculture were important). Village selection was stratified by agro-ecological setting, with 11 pastoral villages and 9 mixed villages selected in total across the nine districts.

A multistage sampling approach was used for the selection of households. Each selected village consisted of two to four sub-villages. Two to three sub-villages were randomly selected for sampling in each village. In each selected sub-village, a central point sampling approach was adopted, where livestock keepers and their animals were invited to a predetermined point within the sub-village. At this central point, a list of the attending households was recorded, and a maximum of ten households selected using a random number generator. From the selected households, a maximum of 10 cattle, 10 sheep and 10 goats were sampled. A target of at least 5 juvenile animals, including 2 juvenile males and 5 adult animals, including 2 adult males were selected at random per species, per household.

### Subpopulation definitions

For this analysis, all samples were allocated to one of two subpopulations: pastoralist, or non-pastoralist. Pastoralist was defined as all animals sampled in pastoral agro-ecological settings in the BacZoo and SEEDZ studies. Non-pastoralist was defined as all animals sampled in peri-urban or agro-pastoral agro-ecological settings in the BacZoo study, or mixed agro-ecological settings in the SEEDZ study.

### Animal-level data and sample collection

Both studies collected individual animal-level data including species, sex, age group (adult or juvenile: where adult was classified as the presence of permanent incisors), breed (indigenous, European-breed, or cross), and brucellosis vaccination status. Up to 10 mL of venous blood was collected into a plain vacutainer (BD, Franklin Lakes, NJ, USA) from all selected livestock. Blood samples were allowed to clot and were centrifuged at 1300–1500 g for 10 minutes. Serum was aliquoted into sterile sample tubes in the field and stored at 4°C in the field before transfer to longer term -80°C storage. All sera were tested by both RBT and cELISA.

### Rose Bengal plate test

All cattle, sheep, and goat sera were tested by the RBT at the field site or at the Kilimanjaro Clinical Research Institute (KCRI) laboratory, Tanzania. All RBT testing was performed following standard protocols [20] and using standardised antigen (RAA0054, APHA, Weybridge, UK). Cattle samples were tested with a 1:1 serum to antigen ratio, whereas sheep and goat samples were tested with a 3:1 serum to antigen ratio (modified RBT) to improve diagnostic sensitivity [48]. Four minutes after mixing the serum with the antigen, any sample that showed visible clumping was classified as positive.

### Competitive ELISA

All livestock sera testing for both studies was performed using the COMPELISA kit (APHA Scientific, Weybridge, UK), following the kit instructions. cELISA testing for the BacZoo study was performed at the APHA, UK, on serum aliquots that had been heat treated at 56°C for two hours. Testing by cELISA for the SEEDZ study was conducted at KCRI on non-heat treated serum aliquots. In all cases, 60% of the mean of the optical density of four conjugate control wells was used as the positive/negative cut-off value for each test plate. Sera with an optical density value equal to, or less than, the cut-off value for each test plate were classified as positive [49].

### Bayesian Hui-Walter model

A Bayesian adaptation of the no gold standard Hui-Walter model [13,26,50] was implemented separately for cattle, sheep, and goat data. RBT and cELISA test outcome probabilities, conditional on an unknown livestock *Brucella* spp. exposure status, were specified using prior information on the sensitivity (Se) and specificity (Sp) of the two diagnostic tests and the seroprevalence (p) of *Brucella* spp. exposure in two subpopulations [27,50]. For this analysis, RBT and cELISA test results were specified as either positive or negative, and all tested animals were classified as originating from one of two subpopulations: pastoralist, or non-pastoralist. The model assumes that for the *i*th subpopulation the counts (O_i_) with each combination of test results (+/+; +/-; -/+; -/-) follow a multinomial distribution [13,26,50]:

$$O_{i}|{Se}_{j},{Sp}_{j},p_{i}∼Multinomial({Pr}_{i},n_{i})\;for\;i=1,2,\ldots ,S\;and\;j=1,2,\ldots ,T$$ (1)

Where Pr_i_ is a vector of probabilities of observing the four combinations of diagnostic test results for the *i*th subpopulation. n_i_ is the total number of observations of the *i*th subpopulation, S is the number of different subpopulations and T is the number of diagnostic tests.

The four equations to determine the probability of observing each combination of diagnostic test outcomes in the *i*th subpopulation are given below [13,26,50]:

$$\begin{array}{l} \mathrm{Pr}\left(T_{1}+,T_{2}+\right)=(\left({Se}_{1}*{Se}_{2}\right))*p_{i}+(\left(1-{Sp}_{1})*(1-{Sp}_{2}\right))*(1-p_{i})\\ \mathrm{Pr}\left(T_{1}+,T_{2}-\right)=\left({Se}_{1}*(1-{Se}_{2}\right))*p_{i}+((1-{Sp}_{1})*{Sp}_{2})*(1-p_{i})\\ \mathrm{Pr}\left(T_{1}-,T_{2}+\right)=((1-{Se}_{1})*{Se}_{2})*p_{i}+({Sp}_{1}*(1-{Sp}_{2}))*(1-p_{i})\\ \;\mathrm{Pr}\left(T_{1}-,T_{2}-\right)=(\left(1-{Se}_{1})*(1-{Se}_{2}\right))*p_{i}+({Sp}_{1}*{Sp}_{2})*(1-p_{i}) \end{array}$$ (2)

Where Pr is the probability of observing the specific combination of diagnostic test outcomes (+/+; +/-; -/+; -/-). T+ is diagnostic test positive and T- is diagnostic test negative, _1_ represents the RBT, _2_ represents the cELISA and p_i_ represents infection prevalence in the *i*th subpopulation.

Series and parallel diagnostic testing approaches were specified in the model based on posterior estimates for sensitivity and specificity for each test according to the following equations:

$$\begin{array}{c} Series\;Se={Se}_{1}*{Se}_{2}\\ Series\;Sp=1-(1-{Sp}_{1})*(1-{Sp}_{2})\\ Parallel\;Se=1-(1-{Se}_{1})*(1-{Se}_{2})\\ Parallel\;Sp={Sp}_{1}*{Sp}_{2} \end{array}$$ (3)

The series diagnostic testing approach tested all samples by RBT and tested all RBT positive samples by cELISA. RBT positive samples confirmed by cELISA positive were considered series test positive. The parallel approach tested all samples by RBT and cELISA, a sample positive by either RBT or cELISA was considered a parallel test positive.

The positive predictive value (PPV) and negative predictive value (NPV) of the RBT only, cELISA only, both tests in series, and both tests in parallel were calculated for pastoralist and non-pastoralist subpopulations in each livestock species model. PPV and NPV were estimated using the sensitivity (Se), specificity (Sp), and subpopulation seroprevalence (p) median posterior estimates [51]:

$$\begin{array}{l} \;PPV=\frac{Se*p}{\left(Se*p)+(1-Sp)*(1-p\right)}\\ NPV=\frac{Sp*(1-p)}{(Sp*(1-p))+(1-Se)*p} \end{array}$$ (4)

### Model assumptions

There are three core assumptions of the Hui-Walter model [25]. The first is that prevalence is different between the subpopulations. There is evidence that *Brucella* spp. seroprevalence is different between predominantly pastoralist communities and other livestock-keeping communities [9], and this difference has also been reported in Tanzania [52,53]. Therefore, there was no reason to believe that this model assumption had been violated.

The second model assumption is that the diagnostic tests perform the same across subpopulations (i.e., have an equivalent sensitivity and specificity). This assumption was considered not to be violated for reasons including: livestock species in both the pastoralist and non-pastoralist subpopulations were largely of the same indigenous or mixed breed; livestock samples from the BacZoo and SEEDZ studies combined were collected over a period spanning two and a half years, helping to account for potential variation in test performance caused by seasonality; and although the laboratories for diagnostic testing and heat treatment of some samples differed between the studies, all samples were tested by RBT and cELISA using common testing protocols and reagents.

The third assumption is that the diagnostic tests included in the model are conditionally independent. That is, the tests perform independently, conditional on an animal’s true disease, or exposure, status [27]. For example, when an animal’s true exposure status is known, conditional independence means that the probability of one test outcome is not affected by knowledge of the other test result [26]. The RBT and cELISA are both serological tests that detect an antibody response to *Brucella* spp. exposure. The RBT utilises an antigen consisting of killed whole *Brucella* cells extracted from smooth *B*. *abortus* [15,20]. Whereas, the cELISA used in these analyses utilises purified smooth lipopolysaccharide antigen extracted from *B*. *melitensis* [54]. Although these tests do differ in the method of antibody detection used, it was conservatively assumed that the model assumption of conditional independence could not be met. Therefore, the model was extended to include conditional dependence, using a covariance parameterisation as described by Vacek [26,55]:

$$\begin{array}{l} \mathrm{Pr}\left(T_{1}+,T_{2}+\right)=(({Se}_{1}*{Se}_{2})+covDp)*p_{i}+((\left(1-{Sp}_{1})*(1-{Sp}_{2}\right))+covDn)*(1-p_{i})\\ \mathrm{Pr}\left(T_{1}+,T_{2}-\right)=((\left({Se}_{1})*(1-{Se}_{2}\right))-covDp)*p_{i}+(((1-{Sp}_{1})*{Sp}_{2})-covDn)*(1-p_{i})\\ \mathrm{Pr}\left(T_{1}-,T_{2}+\right)=((1-{Se}_{1})*{Se}_{2})-covDp)*p_{i}+(({Sp}_{1}*(1-{Sp}_{2}))-covDn)*(1-p_{i})\\ \mathrm{Pr}\left(T_{1}-,T_{2}-\right)=((1-{Se}_{1})*(1-{Se}_{2})+covDp)*p_{i}+(({Sp}_{1}*{Sp}_{2})+covDn)*(1-p_{i}) \end{array}$$ (5)

Where covDp and covDn represent the covariance between the diagnostic tests when an animal is test positive or test negative, respectively.

Species models without covariance were also investigated. Deviance information criterion (DIC) values were compared for species models with and without the covariance parameterisation (see S1 Table).

### Prior distributions

A literature search was performed for publications evaluating the RBT (modified RBT for sheep and goats) or APHA cELISA test sensitivity and specificity in cattle, sheep, and goats. Articles meeting the following criteria were selected to inform prior sensitivity and specificity estimates: published in the last twenty years (1999 to 2019); study sample size clearly indicated; and using culture positive animals, a combination of serology or PCR and culture positive animals, vaccinated animals, experimentally infected animals, suspect exposed animals, or latent class analysis as a method to estimate test performance. For cattle, only studies reporting data from Africa were considered. Equivalent studies in Africa for sheep and goats were limited, therefore studies from varied geographic locations informed these prior estimates and data from sheep and goat populations were combined. RBT and cELISA sensitivity and specificity estimates from the selected literature were summarised by calculating the overall mean weighted by study sample size. Details of each study selected to inform sensitivity and specificity prior estimates for the RBT and cELISA for cattle, sheep and goats are given in S2 and S3 Tables.

Priors assumed a beta distribution with shape parameters (α, β). The prior beta distributions for RBT and cELISA sensitivity and specificity parameters were calculated using the function *epi*.*betabuster* in the R package *epiR* [56]. For each sensitivity or specificity prior, a beta distribution was generated using the weighted mean value from the selected literature (see S2 and S3 Tables) and assuming a value greater than 0.500 with 95% confidence. The sensitivity and specificity priors generated are given in Table 1.

**Table 1:** Prior beta distribution shape parameters (α, β) for sensitivity and specificity of the RBT and cELISA for cattle, sheep and goats.

Test parameter		Beta distribution (α, β)	
		Cattle	Sheep & Goats
RBT	Se	(6.42, 2.02)	(5.40, 1.49)
	Sp	(4.62, 1.13)	(5.13, 1.36)
cELISA	Se	(4.52, 1.09)	(4.43, 1.05)
	Sp	(4.40, 1.03)	(4.34, 1.01)

RBT is the Rose Bengal plate test. For sheep and goat models, RBT refers to the modified RBT 3:1 serum to antigen ratio. cELISA refers to the Animal and Plant Health Agency, UK, competitive enzyme-linked immunosorbent assay. Se is sensitivity and Sp is specificity.

In all models, a vague uniform prior was specified (0, 0.49) for seroprevalence. These priors encompassed the range of seroprevalence estimates in the literature for Tanzania (e.g. [57–59]) and allowed exploration of the wider parameter space.

Uniform prior distributions for the two covariance variables (γ _Se_ and γ _Sp_) were specified using the maximum and minimum limits given below [27,50,60] in all models:

$$\begin{array}{c} ({Se}_{1}-1)(1-{Se}_{2})\leq \gamma_{Se}\leq min({Se}_{1},{Se}_{2})-{Se}_{1}{Se}_{2}\\ ({Sp}_{1}-1)(1-{Sp}_{2})\leq \gamma_{Sp}\leq min({Sp}_{1},{Sp}_{2})-{Sp}_{1}{Sp}_{2} \end{array}$$ (6)

A sensitivity analysis was performed to compare the posterior distributions generated using the above defined literature informed priors and vague uniform (1, 1) priors assigned to sensitivity and specificity test parameters. Plots comparing species model posterior estimates and Bayesian credibility intervals (BCI) generated under the literature informed priors and vague priors are shown in S1–S3 Figs.

### Model implementation

All models were implemented with JAGS [61] in R software version 3.6.1 [62], using the *rjags* R package [63]. Three Markov Chain Monte Carlo (MCMC) chains with different initial starting values were used for each model. The first 50,000 iterations were considered burn-in and discarded. Another 250,000 iterations were run per chain. Of these, every 100^th^ iteration per chain, totaling 7,500 iterations, was used to estimate the posterior distribution. Median posterior and associated 95% BCI estimates were reported. Model code is given in S2 Appendix.

### Model diagnostics

MCMC chain convergence was assessed using the Gelman-Rubin potential scale reduction factor and by visual inspection of Gelman-Rubin, density and trace plots for each parameter. Model diagnostics and visualisations were performed using the *coda* R package [64].

## Results

**Fig 1:**
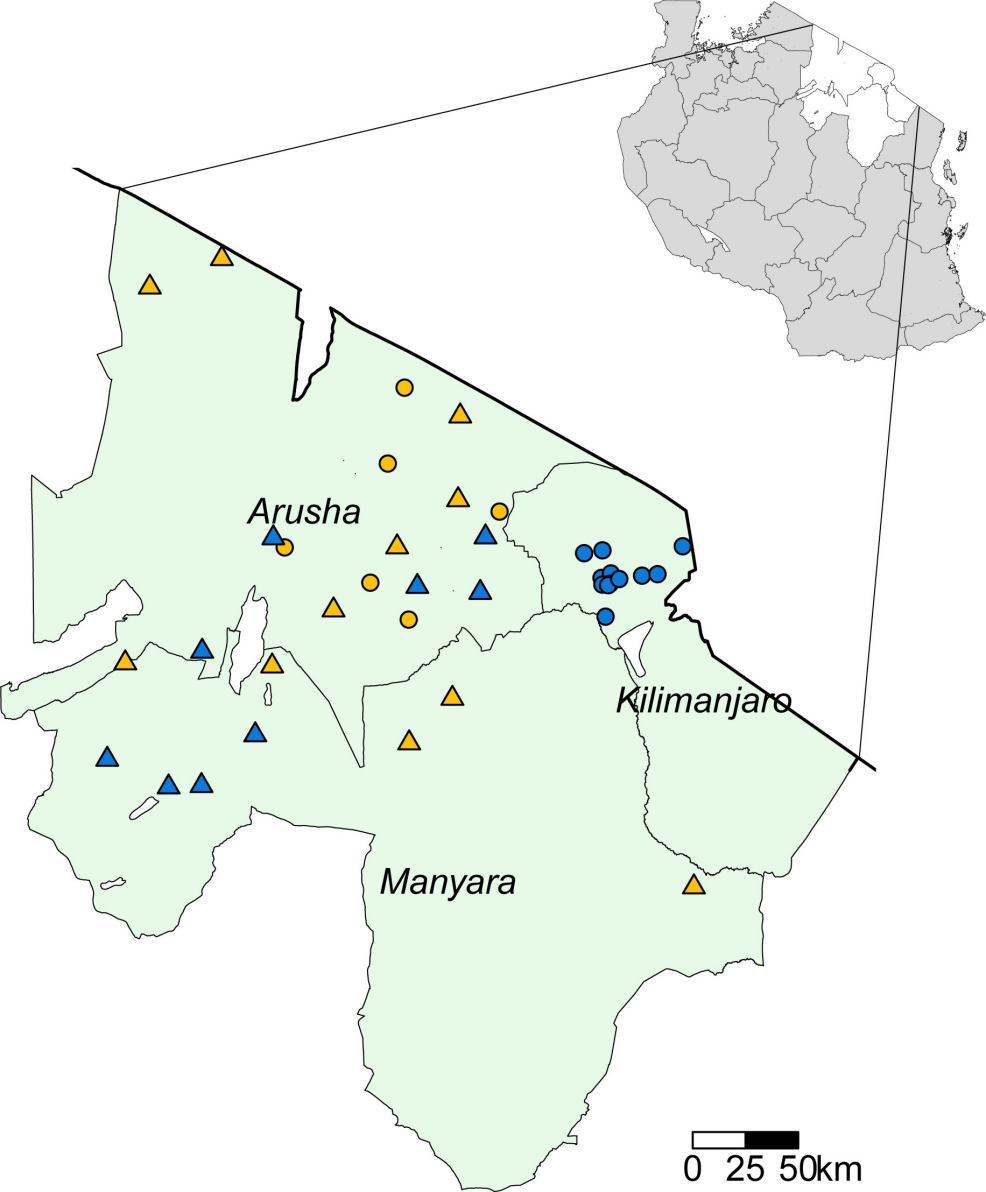
Livestock sampling locations in Arusha, Kilimanjaro, and Manyara Regions (green shading), Tanzania. Solid black line represents the border with Kenya. Circles show the villages sampled for the BacZoo study between 2013 and 2015. Triangles show the villages sampled for the SEEDZ study in 2016. Yellow represents pastoral villages and blue represents non-pastoral villages sampled. In the top right insert, white polygons show Arusha, Kilimanjaro and Manyara Region locations within an outline map of Tanzania (grey shading). Map created using R software 3.6.1 [62] and the tmap R package [65]. Administrative boundary shapefile sourced from the Tanzania National Bureau of Statistics, https://www.nbs.go.tz/index.php/en/census-surveys/population-and-housing-census/172-2012-phc-shapefiles-level-one-and-two [66].

A total of 3467 cattle, 2508 sheep, and 3166 goats had a blood sample collected and tested by both the RBT and the cELISA. Diagnostic test results for pastoralist and non-pastoralist subpopulations are given in Table 2. Contingency tables comparing the RBT and cELISA raw data test outcomes are shown in S4 Table. Cattle and goat samples were collected from 38 villages, 17 (44.7%) of which were from the pastoralist subpopulation. Sheep samples were collected from 35 villages, 17 (48.6%) of which were from the pastoralist subpopulation. The distribution of villages sampled in the two studies is shown in Fig 1.

**Table 2:** RBT and cELISA test outcomes for cattle, sheep, and goats from pastoralist and non-pastoralist subpopulations.

Livestock species	Pastoralist subpopulation		Non-pastoralist subpopulation	
	RBT n/N (%)	cELISA n/N (%)	RBT n/N (%)	cELISA n/N (%)
Cattle	89/2064 (4.3)	120/2064 (5.8)	12/1403 (0.9)	21/1403 (1.5)
Sheep	36/1739 (2.1)	59/1739 (3.4)	15/769 (2.0)	5/769 (0.7)
Goats	81/1892 (4.3)	96/1892 (5.1)	25/1274 (2.0)	19/1274 (1.5)

Cattle, sheep, and goat data are from the combined BacZoo study conducted 2013 to 2015 in Arusha and Kilimanjaro Regions, and the SEEDZ study conducted in 2016 in Arusha and Manyara Regions of Tanzania. RBT is Rose Bengal plate test. For sheep and goat samples, RBT refers to the modified RBT 3:1 serum to antigen ratio. cELISA is the Animal and Plant Health Agency, UK, competitive enzyme-linked immunosorbent assay. N is total number of samples tested and n is total number of test positive samples.

A summary of cattle, sheep, and goat individual characteristics including sex, age (adult or juvenile), and breed (indigenous, European-breed, or cross) is given in Table 3. No vaccination against *Brucella* spp. was reported for livestock sampled in either study.

**Table 3:** Sex, age and breed characteristics for cattle, sheep, and goat data.

Livestock species	Sex	Age group	Breed		
	Female n/N (%)	Adult n/N (%)	Indigenous n/N (%)	European-breed n/N (%)	Cross n/N (%)
Cattle	2341/3464 (67.6)	2415/3458 (69.8)	3031/3462 (87.5)	17/3462 (0.5)	414/3462 (12.0)
Sheep	1910/2507 (76.2)	1887/2505 (75.3)	2498/2507 (99.7)	1/2507 (0.0)	8/2507 (0.3)
Goats	2432/3166 (76.8)	2501/3163 (79.1)	3014/3164 (95.2)	21/3164 (0.7)	129/3164 (4.1)

Cattle, sheep, and goat data are from the combined BacZoo study conducted 2013 to 2015 in Arusha and Kilimanjaro Regions, and the SEEDZ study conducted in 2016 in Arusha and Manyara Regions of Tanzania. N is the total number of animals for which data were available. Age group includes adult versus juvenile.

### Model estimates

**Fig 2:**
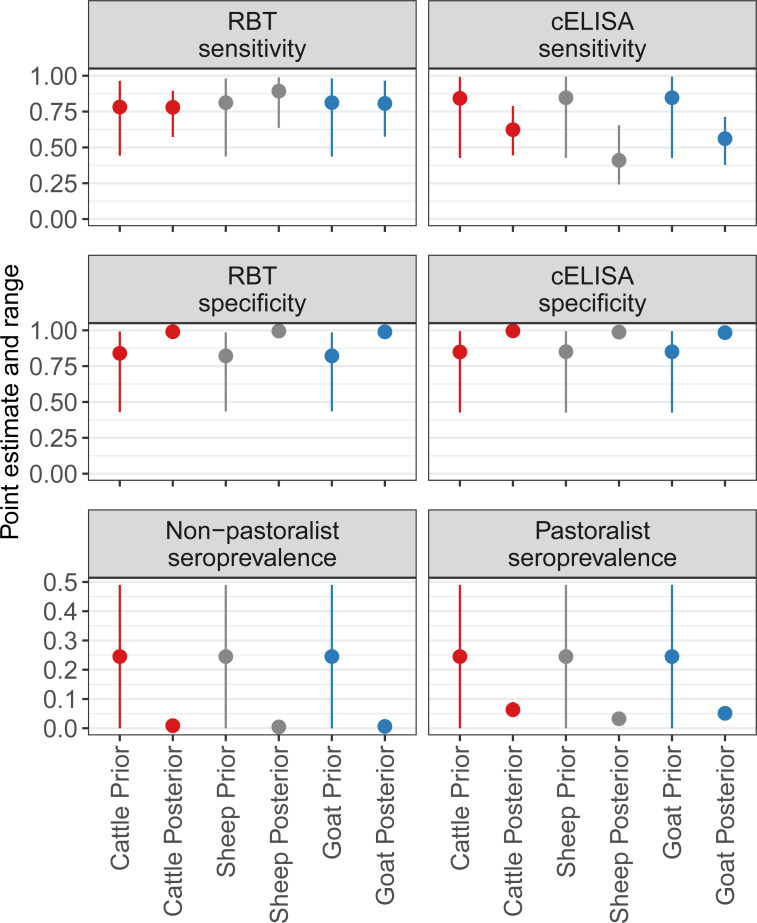
Sensitivity, specificity and seroprevalence prior and posterior point estimates and ranges for species models. Cattle, sheep, and goat data are from the combined BacZoo study conducted 2013 to 2015 in Arusha and Kilimanjaro Regions, and the SEEDZ study conducted in 2016 in Arusha and Manyara Regions of Tanzania. Point estimate refers to beta distribution mode for priors, and the median estimate for posteriors. Range refers to the beta distribution range for priors, and 95% Bayesian credibility interval for posteriors. RBT is Rose Bengal plate test. For sheep and goat models, RBT refers to the modified RBT 3:1 serum to antigen ratio. cELISA is the Animal and Plant Health Agency, UK, competitive enzyme-linked immunosorbent assay.

**Fig 3:**
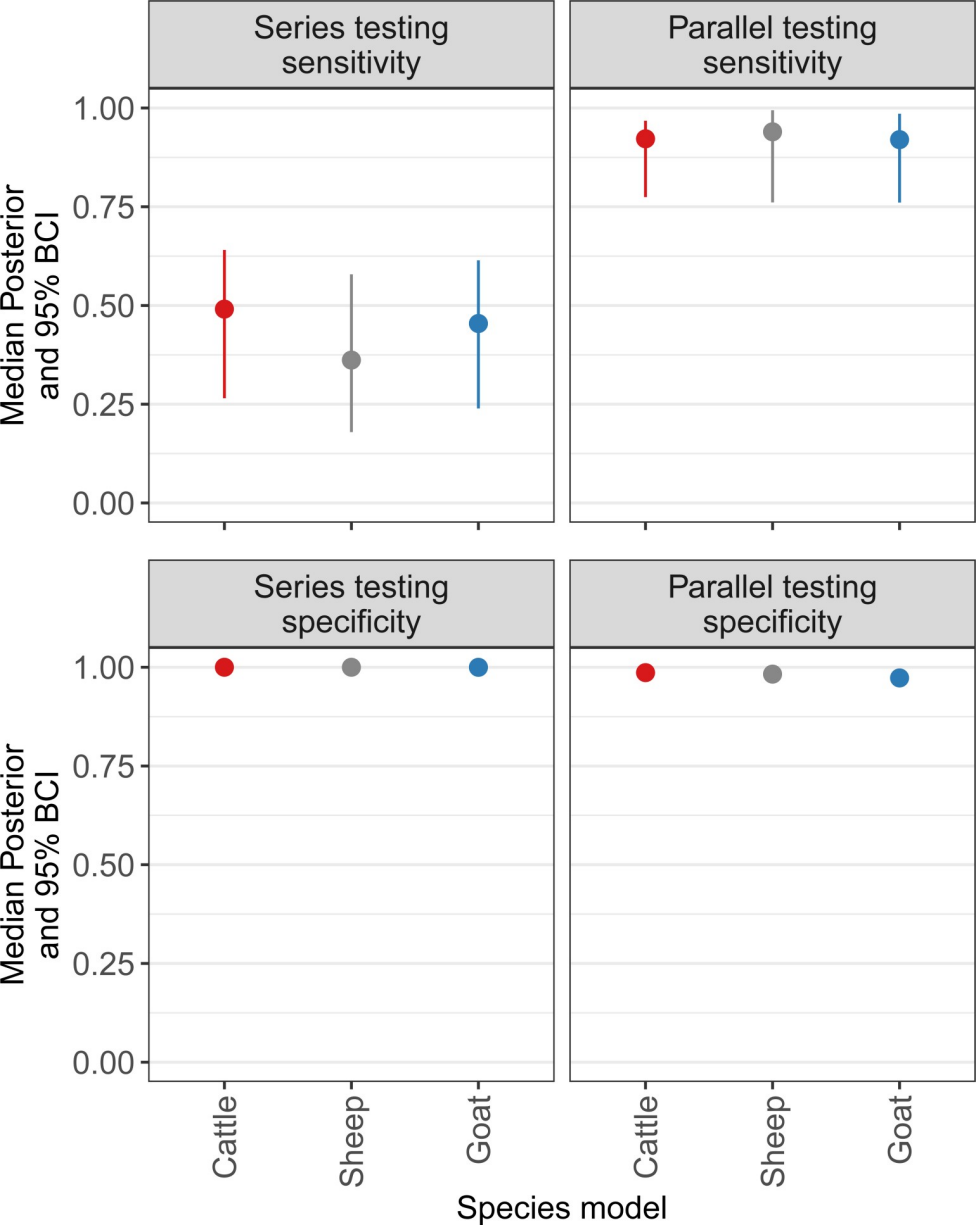
Series and parallel sensitivity and specificity median posterior and 95% BCI estimates for species models. Cattle, sheep, and goat data are from the combined BacZoo study conducted 2013 to 2015 in Arusha and Kilimanjaro Regions, and the SEEDZ study conducted in 2016 in Arusha and Manyara Regions of Tanzania. Series and parallel estimates refer to diagnostic testing using the Rose Bengal plate test (RBT) and the Animal and Plant Health Agency, UK, competitive enzyme-linked immunosorbent assay (cELISA). For sheep and goat models, the modified RBT 3:1 serum to antigen ratio was used. BCI is Bayesian credibility interval.

The test sensitivity, specificity and seroprevalence estimates (including median and 95% BCI) from the cattle, sheep, and goat models are given in Table 4 and plotted for comparison in Fig 2. All species models had a Gelman-Rubin scale reduction factor of <1.1 and showed satisfactory convergence for all model parameters. Density plots for all parameters in each of the species models are given in S4–S6 Figs. Series and parallel diagnostic test approach estimates for each species model are shown in Fig 3. The PPV and NPV estimates for pastoralist and non-pastoralist subpopulations of cattle, sheep, and goats are given in Table 5.

**Table 4:** Median posterior and 95% Bayesian credibility interval parameter estimates for cattle, sheep, and goat models.

Parameter	Species model								
	Cattle			Sheep			Goat		
	Median	2.5% BCI	97.5% BCI	Median	2.5% BCI	97.5% BCI	Median	2.5% BCI	97.5% BCI
Se: RBT	0.779	0.570	0.894	0.893	0.636	0.989	0.807	0.575	0.966
Sp: RBT	0.990	0.980	0.998	0.995	0.985	0.999	0.989	0.980	0.998
Se: cELISA	0.623	0.443	0.790	0.409	0.241	0.644	0.561	0.376	0.713
Sp: cELISA	0.996	0.988	1	0.988	0.981	0.993	0.984	0.974	0.995
Se: series	0.490	0.263	0.641	0.361	0.180	0.573	0.454	0.239	0.614
Sp: series	1	1	1	1	1	1	1	1	1
Se: parallel	0.922	0.772	0.968	0.940	0.760	0.994	0.920	0.760	0.986
Sp: parallel	0.986	0.970	0.997	0.983	0.969	0.991	0.973	0.955	0.992
Seroprevalence: pastoralist subpopulation	0.063	0.045	0.090	0.033	0.018	0.049	0.051	0.034	0.076
Seroprevalence: non-pastoralist subpopulation	0.009	0.001	0.021	0.005	0.000	0.015	0.006	0.000	0.022
covDn	0.002	0.000	0.008	0.002	0.000	0.007	0.009	0.001	0.016
covDp	-0.006	-0.059	0.098	0.000	-0.069	0.078	-0.009	-0.079	0.089

Cattle, sheep, and goat data are from the combined BacZoo study conducted 2013 to 2015 in Arusha and Kilimanjaro Regions, and the SEEDZ study conducted in 2016 in Arusha and Manyara Regions of Tanzania. Se is sensitivity and Sp is specificity. RBT is Rose Bengal plate test. For sheep and goat models, RBT refers to the modified RBT 3:1 serum to antigen ratio. cELISA is the Animal and Plant Health Agency, UK, competitive enzyme-linked immunosorbent assay. covDn is covariance when animal is disease negative. covDp is covariance when animal is disease positive. BCI is Bayesian credibility interval.

**Table 5:** Positive and negative predictive values for RBT only, cELISA only, series, and parallel diagnostic testing approaches for two subpopulations using cattle, sheep, and goat model estimates.

Diagnostic approach	Subpopulation	Species					
		Cattle		Sheep		Goat	
		PPV	NPV	PPV	NPV	PPV	NPV
RBT	Pastoralist	0.840	0.985	0.859	0.996	0.798	0.990
	Non-pastoralist	0.414	0.998	0.473	0.999	0.307	0.999
cELISA	Pastoralist	0.913	0.975	0.538	0.980	0.653	0.977
	Non-pastoralist	0.586	0.997	0.146	0.997	0.175	0.997
Series	Pastoralist	1	0.967	1	0.979	1	0.971
	Non-pastoralist	1	0.995	1	0.997	1	0.997
Parallel	Pastoralist	0.816	0.995	0.654	0.998	0.647	0.996
	Non-pastoralist	0.374	0.999	0.217	1	0.171	1

PPV is positive predictive value and NPV is negative predictive value. RBT is the Rose Bengal plate test. For sheep and goat models, RBT refers to the modified RBT 3:1 serum to antigen ratio. cELISA is the Animal and Plant Health Agency, UK, competitive enzyme-linked immunosorbent assay.

## Discussion

The RBT and cELISA median posterior estimates for specificity were consistently high across species models, whereas median posterior estimates for sensitivity were more variable between tests and across cattle, sheep, and goat models. *Brucella* spp. seroprevalence for all livestock species was estimated to be higher in the pastoralist subpopulation than the non-pastoralist population. The parallel RBT and cELISA testing approach gave the overall best diagnostic test performance estimates across all species models, as compared to series testing. To our knowledge, this is the first study using Bayesian latent class analysis to compare RBT and cELISA test performance to identify *Brucella* spp. exposure in cattle in Tanzania, and the first for sheep and goats in Africa.

There was considerable variation in the median posterior estimates for sensitivity for both diagnostic tests across the different species models. Median posterior estimates for sensitivity were less precise than specificity with wider credibility intervals. The widest BCI was for cELISA sensitivity in sheep. The estimated sensitivity of the RBT was higher than the cELISA in all species models, although there was overlap in the credibility intervals. All species median posterior estimates for sensitivity of the cELISA in this study were lower than the reviewed literature. This difference may be due to a number of reasons, such as the low number of test positive animals in our study. Low seroprevalence of *Brucella* spp. exposure leads to small sample sizes for test positive animals and in scenarios where the test positive sample size is small, the point estimate may be affected and less precise credibility intervals can be expected, particularly for sensitivity [26]. Also, the smaller the difference in seroprevalence between subpopulations, the greater the uncertainty around sensitivity and specificity estimates [26]. In our study, the difference in seroprevalence estimates between subpopulations was variable, with the smallest difference between the pastoral and non-pastoral seroprevalences observed for sheep. These are likely some of the factors that led to wider credibility intervals on test sensitivity estimates and in all cases, estimates with wide credibility intervals should be interpreted cautiously.

Sensitivity analyses comparing posterior estimates obtained with the literature informed (final model) priors to equivalent estimates obtained from model runs with vague uniform (1, 1) priors, demonstrated that specificity posterior estimates remained high with vague priors specified (see S1–S3 Figs). However, posterior estimates of sensitivity were less robust to the vague priors. Although posterior estimates of sensitivity were lower with vague priors, credibility intervals did still overlap with those of the literature informed priors (see S1–S3 Figs), suggesting that the literature informed priors were not overwhelming the data and driving the posterior estimates.

Median posterior estimates for seroprevalence in the pastoralist subpopulation for cattle, sheep, and goats were 0.063, 0.033, and 0.051, respectively. In the non-pastoralist population, estimated seroprevalence was less than 0.01 for all livestock species. These seroprevalence estimates are broadly consistent with findings reported in comparable studies in Tanzania [52,53,57–59,67–71]. Seroprevalence in Tanzania has been reported up to 21.5% for cattle, 7.7% for sheep, and 11.5% for goats [58,59]. Increased livestock seroprevalence in pastoral settings has been reported previously in Tanzania [52,53], and may be due to specific livestock keeping practices found in pastoralist communities, such as large herd or flock sizes and the mixing of livestock species [9].

The performance of the series diagnostic testing approach (i.e., testing all animals by RBT, with RBT positive animals confirmed by cELISA) and the parallel diagnostic testing approach (i.e., testing all animals by both RBT and cELISA) were also evaluated. The median posterior estimates for specificity when testing in series were consistently high for all species models. However, median posterior estimates for sensitivity were substantially reduced, as compared to the parallel testing approach. Running the two tests in parallel gave improved median estimates for sensitivity and median estimates for specificity were only marginally reduced in each species model, as compared to series testing. The median posterior estimates for sensitivity when testing in parallel were 0.922, 0.940, and 0.920 for cattle, sheep, and goat models, respectively. The median posterior estimates for specificity when testing in parallel for all species models were 0.973 or greater. The NPV was higher for parallel as compared to series testing for all species models. The PPV was low for a parallel testing approach, which was to be expected, particularly in the non-pastoralist subpopulation, as the low seroprevalence estimates would have influenced the predictive values. In the context of surveillance for the purpose of generating initial baseline livestock seroprevalence estimates to guide subsequent national prevention and control activities, a high NPV can be important in demonstrating that a test negative animal is truly negative for *Brucella* spp. exposure.

In Tanzania, the national zoonotic disease surveillance guidelines indicate the need for active surveillance of animal brucellosis in the form of mass screening, using bacteriological, molecular or serological diagnostic methods [24]. As of yet, there is no nationally adopted livestock diagnostic testing strategy [23]. The outcomes of this study can therefore be used to guide decision-making regarding a diagnostic testing approach for national active surveillance. Our study results suggest that a testing approach applying RBT and cELISA in parallel for detection of *Brucella* spp. exposure would give the best diagnostic test sensitivity and specificity in cattle, sheep, and goats. Our findings also reinforce the importance of utilising these two tests in combination. A single test approach, particularly in low seroprevalence subpopulations, would compromise the accuracy of *Brucella* spp. exposure estimates. However, testing all animals nationally would be extremely resource intensive. A viable alternative could be testing for *Brucella* spp. exposure at the herd- or flock-level on a predetermined proportion of animals [20]. The identification of one or more seropositive animal would indicate a seropositive herd or flock [3], leading to individual testing of animals in the seropositive herd or flock. Another potential surveillance method at the herd- or flock-level could include sample pooling, whereby a herd or flock are split into subgroups and individual animal serum samples combined for subgroup testing, with subsequent individual animal testing for seropositive subgroups [72]. The pooling of serum samples can reduce surveillance screening time and cost, and potentially improve diagnostic sensitivity and specificity, particularly in low seroprevalence populations [72]. Pooled sampling techniques have been applied to livestock diseases such as bovine viral diarrhoea virus, enzootic bovine leukosis virus, and Q fever [73–75]. To apply pooled sampling to the surveillance of *Brucella* spp. exposure in livestock, a sensitivity analysis is needed to estimate the optimal pool size per herd or flock for the detection of *Brucella* spp. antibodies, as has been performed for the detection of infections such as *Schistosoma japonicum* [76]. It would also be important to generate cost estimates for a pooled approach under varying *Brucella* spp. exposure seroprevalence scenarios.

The Food and Agriculture Organization of the United Nations has developed guidelines for the design of a surveillance system for animal and human brucellosis [77]. These guidelines can be used to form a surveillance strategy for detection of *Brucella* spp. exposures. In Tanzania and similar settings where brucellosis prevention and control programmes are in the development or initial phases, a starting point would be to clearly define the surveillance goals. For example, national livestock surveillance for the purpose of generating robust baseline seroprevalence estimates would be valuable in informing subsequent stages of prevention and control, such as vaccination. Another important step in the design of livestock surveillance would be to review the existing national data, particularly focusing on human infections, so as to initially prioritise high-risk animal to human transmission areas. For example, both *B*. *melitensis* and *B*. *abortus* have been isolated from a pastoralist community in northern Tanzania [44], indicating a priority area for surveillance in cattle, goats, and sheep. Also, the training of local government veterinary representatives in the implementation of the RBT in the field is necessary. Equally, strengthening the capacity for cELISA testing is required, including training of personnel and provision of appropriate equipment at regional government veterinary laboratories. Furthermore, ensuring common diagnostic testing protocols, use of standardised and appropriately sourced and stored reagents that have been subject to national quality assurance and control, and providing appropriate sample transport and storage conditions from sample collection sites, are all necessary in generating robust data. Accomplishing these steps would help facilitate livestock screening for *Brucella* spp. exposure by RBT in the field, with complementary parallel testing by cELISA in selected laboratories. The provision of capacity to implement these serological tests would also improve the ability to implement diagnostic testing for other infectious diseases.

It is important to consider that there are limitations associated with the use of latent class models, particularly regarding the core model assumptions. The model assumption that diagnostic test performance is the same across subpopulations is difficult to meet [15]. In our study, the assumption of consistent diagnostic test performance was considered met as livestock species sampled, timing of sampling and diagnostic testing procedures were similar across subpopulations and cross-sectional studies. However, there are various other factors that may influence the performance of diagnostic tests between subpopulations. For example, as *Brucella* spp. seroprevalence is variable in different subpopulations, it is possible that the prevalence of pathogens causing serological cross-reactions may vary too, differentially influencing the performance of serological tests. The impact of not sufficiently meeting this model assumption merits further investigation. Another assumption of the Hui-Walter model that is often difficult to satisfy is that of conditional independence between diagnostic tests. In our study this assumption could not be met and so a covariance parameterisation accounting for conditional dependence was included for each species model. Toft *et al*. emphasize that when using latent class analysis the diagnostic tests evaluated should measure different physiological responses wherever possible, so that the central assumption of conditional independence can be met [26]. Therefore, implementation of latent class analysis evaluating RBT or cELISA against PCR for example, could be further investigated in this setting. Finally, the implementation of these analyses in low seroprevalence settings with low numbers of test positive animals leads to challenges in estimating test performance, particularly test sensitivity. At least 300 test positive animals has been suggested as a sufficient sample size [15], yet in low prevalence contexts such data may be difficult to attain for brucellosis. In this study, the wide credibility intervals on sensitivity parameter estimates likely reflects the limited data informing these estimates. Further data generation to improve the precision of these performance estimates in the northern Tanzania setting, e.g., through targeted sampling of seropositive animals, would be useful in further evaluating test sensitivity estimates.

## Conclusion

Our study estimated median posterior estimates for RBT and cELISA sensitivity to vary between cattle, sheep, and goat models. Median estimated specificity for the RBT and cELISA was consistently high across species models. All livestock species median estimates for seroprevalence were higher in the pastoralist subpopulation than the non-pastoralist subpopulation, providing further evidence that the burden of brucellosis is likely to be greater in pastoralist communities. A parallel RBT and cELISA testing approach resulted in the best diagnostic test performance in all species models, as compared to a series approach. There is a need to evaluate strategies for implementing parallel testing at the herd- and flock-level. Overall, these results are important in assisting in the development of a national livestock surveillance strategy, that can generate robust *Brucella* spp. exposure estimates for Tanzania and could be applied to similar settings in sub-Saharan Africa. The implementation of a surveillance strategy utilising a standardised, locally evaluated diagnostic testing approach is an important step towards the prevention and control of brucellosis in both animals and humans.

## Supporting information

S1 Appendix Bacterial zoonoses (BacZoo) study methods. (PDF)

S2 Appendix Bayesian latent class model code. (PDF)

S1 Table Deviance information criterion values for the cattle, sheep and goat models with literature informed and vague uniform priors for test sensitivity and specificity, with and without the covariance parameterisation. (PDF)

S2 Table Mean sensitivity and specificity estimates for the RBT and cELISA in cattle taken from a literature search of scientific publications based in Africa from 1999–2019. (PDF)

S3 Table Mean sensitivity and specificity estimates for the RBT and cELISA in sheep and goats taken from a literature search of global scientific publications from 1999–2019. (PDF)

S4 Table Contingency tables comparing RBT and cELISA test results for cattle, sheep, and goat data. (PDF)

S1 Fig Sensitivity, specificity and seroprevalence point estimates and ranges for literature informed and vague uniform cattle model priors and posteriors. (PDF)

S2 Fig Sensitivity, specificity and seroprevalence point estimates and ranges for literature informed and vague uniform sheep model priors and posteriors. (PDF)

S3 Fig Sensitivity, specificity and seroprevalence point estimates and ranges for literature informed and vague uniform goat model priors and posteriors. (PDF)

S4 Fig Density plots of cattle model parameters. (PDF)

S5 Fig Density plots of sheep model parameters. (PDF)

S6 Fig Density plots of goat model parameters. (PDF)
