# Supplementary material for: Latent class evaluation of the performance of serological tests for exposure to Brucella spp. in cattle, sheep, and goats in Tanzania
Source: PLoS Negl Trop Dis. 2021 Aug 24;15(8):e0009630. doi: 10.1371/journal.pntd.0009630 (PMC8384210; doi:10.1371/journal.pntd.0009630)
Supplement: S1 Appendix — (PDF) [file pntd.0009630.s001.pdf]

S1 Appendix. Bacterial zoonoses (BacZoo) study methods.

Study area

A cross-sectional survey of livestock owning households was conducted in the Kilimanjaro & Arusha regions in northern Tanzania between September 2013 and March 2015. Six districts (Hai, Longido, Monduli, Moshi Municipal, Moshi Rural and Rombo) were selected to encompass a range of livestock keeping systems including peri-urban, agro-pastoral and pastoral systems, accounting for distance to study laboratory facilities and engagement of district level administrative representatives.

A list of all wards within each district at the time of the study was obtained, using data from the 2002 census, 2012 census [1], and consultation with administrative representatives. In each district, wards were prospectively classified into three study defined agro-ecological settings – peri-urban, agro-pastoral and pastoral – for stratification as follows. All wards were classified as Rural or Urban based on data recorded in the 2002 census. Information on the predominant communities present within all wards was obtained through direct communication with district level administrative representatives. Peri-urban wards were defined as urban wards within the Hai, Moshi Municipal, Moshi Rural and Rombo Districts of Kilimanjaro Region. Agro-pastoral wards were defined as rural wards within the Hai, Moshi Municipal, Moshi Rural and Rombo Districts of Kilimanjaro region that did not contain a substantial population of pastoralist livestock keepers. Pastoral wards were defined as rural wards within Longido & Monduli Districts of the Arusha Region that included a substantial population of pastoralist livestock keepers. Wards not meeting the classification criteria for any of the three study settings were not included in the study.

Multi-stage sampling

A multistage sampling approach was adopted to select wards, village, sub-village and livestock owning households for inclusion in the study. Six wards were selected at random from each agro-ecological setting to give a total of 18 study wards.

Within selected wards a list of all villages or sub-villages (depending on the smallest unit applicable to each ward), was created in consultation with administrative representatives and one village or sub-village was randomly selected for inclusion using a random number generator. Within the selected village or sub-village households were randomly selected from a list of livestock owning households (defined as households owning one or more cattle, sheep and/or goats) generated through consultation with local community leaders in each village. A minimum of five households were recruited in each village or sub-village to achieve a target village sample size of 50 individuals from each livestock species (cattle, sheep and goats). Livestock ownership patterns varied considerably between study wards. In pastoral wards, the target sample size was typically achieved by sampling at five households in each village. In peri-urban wards and agro-pastoral, additional households were recruited until either the target number of animals were sampled or a maximum time for data collection (two working weeks) within that ward was reached.

Collection of household and animal data

A questionnaire was administered with an adult representative of each household to collect herd-level data including livestock ownership (e.g. species and number of animals kept) and management practices (e.g. grazing, watering and breeding practices) as well as reported

interactions between livestock and wildlife species (e.g. buffalo, wildebeest, rodents). GPS co-ordinates and the altitude of each study household were also recorded.

At each household, up to 15 cattle, 15 sheep and 15 goats were selected for sampling. In households with more than 15 cattle, sheep or goats, adult female animals of each species were prioritised for sampling. Individual animal level data was collected including sex (female, male, castrated), breed (indigenous, exotic or cross-breed), body condition score (5-point scale) [2–4], reproductive history, milk production (where applicable), clinical history and reported brucellosis vaccination status. Animal age class was determined using dentition eruption patterns [5–7]. For the purpose of this analysis, animals were classified as adult or juvenile, animals with any permanent incisors were classified as adult.

Collection and processing of blood samples
Venous blood samples (up to 10 mL) were taken from all selected cattle, sheep, and goats following questionnaire administration. Blood samples were collected into plain vacutainers (BD, Franklin Lakes, NJ, USA), inverted and left to clot before being centrifuged at 1300-1500 g for 10 minutes on the day of collection. Serum was separated into sterile sample tubes, stored at 4 °C in a mobile refrigerator in the field for up to 72 hours and then transferred to longer term storage at -80 °C. Serum were heat treated at 56 °C for two hours prior to import into the UK for cELISA testing.

References

1. NBS. The United Republic of Tanzania 2012 Population and Housing Census. Dar es Salaam, United Republic of Tanzania; 2013.
2. Mongeon M, Wright T, French J. Body condition scoring of dairy cattle. In: Ministry of Agriculture, Food and Rural Affairs, Ontario, Canada [Internet]. 2020 [cited 9 Sep 2020]. Available: https://www.ontario.ca/page/body-condition-scoring-dairy-cattle
3. Government of Canada. Body Condition Scoring Dairy Goats. In: Ministry of Agriculture, Food and Rural Affairs, Ontario, Canada [Internet]. 2020 [cited 9 Sep 2020]. Available: http://www.omafra.gov.on.ca/english/livestock/goat/news/dgg1708a5.htm
4. Curnow M. Condition scoring of sheep. In: Department of Primary Industries and Regional Development, Government of Australia [Internet]. 2018 [cited 9 Sep 2020]. Available: https://www.agric.wa.gov.au/management-reproduction/condition-scoring-sheep
5. Casburn G. Primefact: How to tell the age of sheep. In: Department of Primary Industries, NSW Government [Internet]. 2016 [cited 9 Sep 2020]. Available: https://www.dpi.nsw.gov.au/animals-and-livestock/sheep/management/general-information/age
6. Mitchell T. Agfact A7.2.2 How to tell the age of goats. In: NSW Agriculture [Internet]. 2003 [cited 9 Sep 2020]. Available: https://www.dpi.nsw.gov.au/__data/assets/pdf_file/0007/178477/age-of-goats.pdf
7. Herzog CM, de Glanville WA, Willett BJ, Kibona TJ, Cattadori IM, Kapur V, et al. Pastoral production is associated with increased *peste des petits ruminants* seroprevalence in northern Tanzania across sheep, goats and cattle. Epidemiol Infect. 2019;147: e242. doi: 10.1017/S0950268819001262.
