# Supplementary material for: Latent class evaluation of the performance of serological tests for exposure to Brucella spp. in cattle, sheep, and goats in Tanzania
Source: PLoS Negl Trop Dis. 2021 Aug 24;15(8):e0009630. doi: 10.1371/journal.pntd.0009630 (PMC8384210; doi:10.1371/journal.pntd.0009630)
Supplement: S2 Appendix — (PDF) [file pntd.0009630.s002.pdf]

S2 Appendix. Bayesian latent class model code.

```
# Set up data for JAGS
df <- read.csv("Bodenhametal2021_PLOSNTDs_BrucellaLCA_SPECIESData_2021-02-16.csv")

# keep only columns: test1, test2, subpopulation (P = Pastoralist, O = Non-pastoralist)
df2 <- df %>% select (rbt_result, celisa_result, subpop)
colnames(df2) <- c("rbt","celisa","subpop")

pop <- t(matrix(with(df2, table(rbt, celisa, subpop), dnn=c("rbt", "celisa", "subpop")), 4,2))
n.pop = 2
n = apply(pop,1,sum)
colnames(pop) <- c(" -- ", " +- ", " -+ ", " ++ ")

# JAGS model
# [1] is RBT
# [2] is cELISA
# Tests in order  -- / +- / -+ / ++

cat("model{
   for (i in 1:n.pop){
   pop[i, 1:4] ~ dmulti(par[i, 1:4], n[i])
   p[i] ~ dunif(0, 0.49)
   par[i,4] <- p[i]*   (Se[2] * Se[1] + covDp) + (1-p[i])*((1-Sp[2])*(1-Sp[1]) + covDn) #11
   par[i,3] <- p[i]* ((1-Se[2])* Se[1] - covDp) + (1-p[i])*((Sp[2])*(1-Sp[1]) - covDn) #01
   par[i,2] <- p[i]*   (Se[2] * (1-Se[1]) - covDp) + (1-p[i])*((1-Sp[2])*(Sp[1]) - covDn) #10
   par[i,1] <- p[i]* ((1-Se[2])* (1-Se[1]) + covDp) + (1-p[i])*((Sp[2])*(Sp[1]) + covDn) #00
   }

   ls <- (Se[1]-1)*(1-Se[2])
   us <- min(Se[1],Se[2]) - Se[1]*Se[2]
   lc <- (Sp[1]-1)*(1-Sp[2])
   uc <- min(Sp[1],Sp[2]) - Sp[1]*Sp[2]
   rhoD <- covDp / sqrt(Se[1]*(1-Se[1])*Se[2]*(1-Se[2]))
   rhoDc <- covDn / sqrt(Sp[1]*(1-Sp[1])*Sp[2]*(1-Sp[2]))

   # tests in order  -- / +- / -+ / ++

   # FOR CATTLE MODEL ONLY:
   Se[1] ~ dbeta(6.42,2.02)
   Sp[1] ~ dbeta(4.62,1.13)
   Se[2] ~ dbeta(4.52,1.09)
   Sp[2] ~ dbeta(4.40,1.03)

   # FOR SHEEP & GOAT MODELS:
   Se[1] ~ dbeta(5.40,1.49)
   Sp[1] ~ dbeta(5.13,1.36)
   Se[2] ~ dbeta(4.43,1.05)
   Sp[2] ~ dbeta(4.34,1.01)

   #To get Serial and Parallel Ses and Sps
   Se_series <- Se[1] * Se[2]
```

```
    Se_parallel <- 1 - (1 - Se[1]) * (1 - Se[2])
    Sp_series <- 1 - (1 - Sp[1]) * (1 - Sp[2])
    Sp_parallel <- Sp[1] * Sp[2]
    covDn ~ dunif(lc, uc)
    covDp ~ dunif(ls, us)

    }", file="mod.jag")
```

# Initial values for the three chains

# CATTLE MODEL ONLY:

```
modelInit1 <- list(Se=c(0.4,0.99),  Sp=c(0.7,0.95),  p=c(0,0.1))
modelInit2 <- list(Se=c(0.3,0.98),  Sp=c(0.5,0.9),  p=c(0.01,0.25))
modelInit3 <- list(Se=c(0.2,0.8),  Sp=c(0.3,0.99),  p=c(0.02,0.49))
INI <- list(modelInit1, modelInit2,modelInit3)
```

# FOR SHEEP & GOAT MODELS:

```
modelInit1 <- list(Se=c(0.7,0.99),  Sp=c(0.3,0.99),  p=c(0,0.1))
modelInit2 <- list(Se=c(0.3,0.8),  Sp=c(0.15,0.7),  p=c(0.01,0.25))
modelInit3 <- list(Se=c(0.6,0.95),  Sp=c(0.4,0.90),  p=c(0.02,0.49))
INI <- list(modelInit1, modelInit2,modelInit3)
```

# Compile model components

```
M <- jags.model(data=list(pop=pop,n=n, n.pop=n.pop), inits=INI, n.chains=3, n.adapt= 50000,
file="mod.jag")
```

# Run the model with 50,000 burn-in and a further 250,000 iterations and thinning every 100th iteration

```
R <- coda.samples(M, c("Se", "Sp", "p", "Se_series", "Se_parallel", "Sp_series", "Sp_parallel"),
n.iter=250000, n.thin=100)
```

# Check model deviance information criterion (DIC)

```
dic.samples(M, n.iter=250000, n.thin=100, type="pD")
```

# Check model convergence

```
densityplot(R)
gelman.diag(R, multivariate = FALSE)
gelman.plot(R)
traceplot(R)
```
