# Supplementary material for: Latent class evaluation of the performance of serological tests for exposure to Brucella spp. in cattle, sheep, and goats in Tanzania
Source: PLoS Negl Trop Dis. 2021 Aug 24;15(8):e0009630. doi: 10.1371/journal.pntd.0009630 (PMC8384210; doi:10.1371/journal.pntd.0009630)
Supplement: S1 Table — (PDF) [file pntd.0009630.s003.pdf]

S1 Table. Deviance information criterion values for the cattle, sheep and goat models with literature informed and vague uniform priors for test sensitivity and specificity, with and without the covariance parameterisation.

	**Species model**					
	**Cattle**		**Sheep**		**Goat**	
**Model parameter**	**Literature informed priors**	**Vague priors**	**Literature informed priors**	**Vague priors**	**Literature informed priors**	**Vague priors**
With covariance	34.03	33.87	34.83	33.55	35.22	34.99
Without covariance	34.32	34.07	37.50	35.86	40.61	39.67

Literature informed estimates were generated using a literature search of scientific publications from 1999-2019. Cattle estimates were calculated from studies based in Africa, sheep and goat estimates were calculated from studies from varied geographic locations. Vague priors were specified as dbeta(1,1). DIC is the deviance information criterion.
