# Supplementary material for: Latent class evaluation of the performance of serological tests for exposure to Brucella spp. in cattle, sheep, and goats in Tanzania
Source: PLoS Negl Trop Dis. 2021 Aug 24;15(8):e0009630. doi: 10.1371/journal.pntd.0009630 (PMC8384210; doi:10.1371/journal.pntd.0009630)
Supplement: S2 Table — (PDF) [file pntd.0009630.s004.pdf]

S2 Table. Mean sensitivity and specificity estimates for the RBT and cELISA in cattle taken from a literature search of scientific publications based in Africa from 1999-2019.

Reference	Methods used for test performance estimation	Location	RBT: Se	RBT: Sp	cELISA: Se	cELISA: Sp	Sample size
Muma *et al*., 2007 [1]	LCA vague uniform priors	Zambia	0.910	0.810	-	-	189
Bronsvoort *et al*., 2009 [2]	LCA informative priors from: [3–8]	Cameroon	-	-	0.978	0.987	1375
Matope *et al*., 2011 [9]	LCA vague uniform priors	Zimbabwe	0.947*	0.990*	-	-	876
Sanogo *et al*., 2013 [10]	LCA informative priors from: [11]	Ivory Coast	0.547	0.978	-	-	995
Etman *et al*., 2014 [12]	LCA	Egypt	0.961	0.993	0.971	1	400
Getachew *et al*., 2016 [13]	LCA informative priors from: [14,15]	Ethiopia	0.896	0.845	-	-	278
Chisi *et al*., 2017 [16]	*Se:* Culture positive animals (naturally infected)<br>*Sp*: *Brucella* free animals	South Africa	0.958	1	-	-	232
Pfukenyi *et al*., 2020 [17]	LCA informative priors from: [2,9,18]	Zimbabwe & Botswana	0.897*	0.969*	-	-	1792
Weighted mean			0.842	0.965	0.976	0.990	

Se is sensitivity and Sp is specificity. LCA is latent class analysis. RBT is the Rose Bengal plate test. cELISA is the Animal and Plant Health Agency, UK, competitive enzyme-linked immunosorbent assay. * denotes median sensitivity and specificity. Weighted mean is the mean value weighted by sample size.

References

1. Muma JB, Toft N, Oloya J, Lund a., Nielsen K, Samui K, et al. Evaluation of three serological tests for brucellosis in naturally infected cattle using latent class analysis. Vet Microbiol. 2007;125: 187–192. doi: 10.1016/j.vetmic.2007.05.012.
2. Bronsvoort BM de C, Koterwas B, Land F, Handel IG, Tucker J, Morgan KL, et al. Comparison of a flow assay for brucellosis antibodies with the reference cELISA test in West African *Bos indicus*. PLoS One. 2009;4: e5221. doi: 10.1371/journal.pone.0005221.
3. Fosgate GT, Adesiyun AA, Hird DW, Johnson WO, Hietala SK, Schurig GG, et al. Estimation of receiver-operating characteristic curves to determine accuracy of a competitive enzyme-linked immunosorbent assay for the serodiagnosis of Brucella infection in domestic water buffalo (Bubalus bubalis) and cattle. Am J Vet Res. 2003;64: 57–64. doi: 10.2460/ajvr.2003.64.57.
4. Gall D, Colling A, Marino O, Moreno E, Nielsen K, Perez B, et al. Enzyme immunoassays for serological diagnosis of bovine brucellosis: A trial in Latin America. Clin Diagn Lab Immunol. 1998;5: 654–61. Available: http://www.ncbi.nlm.nih.gov/pubmed/9729532%0Ahttp://www.pubmedcentral.nih.gov/articlerender.fcgi?artid=PMC95636
5. McGiven JA, Tucker JD, Perrett LL, Stack JA, Brew SD, MacMillan AP. Validation of FPA and cELISA for the detection of antibodies to Brucella abortus in cattle sera and comparison to SAT, CFT, and iELISA. J Immunol Methods. 2003;278: 171–178. doi: 10.1016/S0022-1759(03)00201-1.
6. Nielsen KH, Kelly L, Gall D, Nicoletti P, Kelly W. Improved competitive enzyme immunoassay for the diagnosis of bovine brucellosis. Vet Immunol Immunopathol. 1995;46: 285–291. doi: 10.1016/0165-2427(94)05361-U.
7. Nielsen K, Kelly L, Gall D, Balsevicius S, Bosse J, Kelly W, et al. Comparison of enzyme immunoassays for the diagnosis of bovine brucellosis. Prev Vet Med. 1996;26: 17–32.
8. Stack JA, Perrett LL, Brew SD, MacMillan AP. Competitive ELISA for bovine brucellosis suitable for testing poor quality samples. Vet Rec. 1999;145: 735–736.
9. Matope G, Muma JB, Toft N, Gori E, Lund A, Nielsen K, et al. Evaluation of sensitivity and specificity of RBT, c-ELISA and fluorescence polarisation assay for diagnosis of brucellosis in cattle using latent class analysis. Vet Immunol Immunopathol. 2011;141: 58–63. doi: 10.1016/j.vetimm.2011.02.005.
10. Sanogo M, Thys E, Achi YL, Fretin D, Michel P, Abatih E, et al. Bayesian estimation of the true prevalence, sensitivity and specificity of the Rose Bengal and indirect ELISA tests in the diagnosis of bovine brucellosis. Vet J. 2013;195: 114–120. doi: 10.1016/j.tvjl.2012.06.007.
11. Nielsen K. Diagnosis of brucellosis by serology. Vet Microbiol. 2002;90: 447–459. doi: 10.1016/s0378-1135(02)00229-8.
12. Etman RH, Barsoum SA, Ibrahim IGA, El-Ashmawy WR, Abou-Gazia KA. Evaluation of efficacy of some serological tests used for diagnosis of brucellosis in cattle in Egypt using latent class analysis. Sokoto J Vet Sci. 2014;12: 1–7. doi: 10.4314/sokjvs.v12i2.1.
13. Getachew T, Getachew G, Sintayehu G, Getenet M, Fasil A. Bayesian Estimation of Sensitivity and Specificity of Rose Bengal, Complement Fixation, and Indirect ELISA Tests for the Diagnosis of Bovine Brucellosis in Ethiopia.

Vet Med Int. 2016;2016: 1–5. doi: 10.1155/2016/8032753.
14. Gall D, Nielsen K. Serological diagnosis of bovine brucellosis: A review of test performance and cost comparison. OIE Rev Sci Tech. 2004;23: 989–1002.
15. Mainar-Jaime RC, Muñoz PM, de Miguel MJ, Grilló MJ, Marín CM, Moriyón I, et al. Specificity dependence between serological tests for diagnosing bovine brucellosis in Brucella-free farms showing false positive serological reactions due to Yersinia enterocolitica O:9. Can Vet J. 2005;46: 913–916.
16. Chisi SL, Marageni Y, Naidoo P, Zulu G, Akol GW, van Heerden H. An evaluation of serological tests in the diagnosis of bovine brucellosis in naturally infected cattle in KwaZulu-Natal province in South Africa. J S Afr Vet Assoc. 2017;88: 1–7. doi: 10.4102/jsava.v88i0.1381.
17. Pfukenyi DM, Meletis E, Modise B, Ndengu M, Kadzviti FW, Dipuo K, et al. Evaluation of the sensitivity and specificity of the lateral flow assay, Rose Bengal test and the complement fixation test for the diagnosis of brucellosis in cattle using Bayesian latent class analysis. Prev Vet Med. 2020;181: 105075. doi: 10.1016/j.prevetmed.2020.105075.
18. Gusi AM, Bertu WJ, Jesús de Miguel M, Dieste-Pérez L, Smits HL, Ocholi RA, et al. Comparative performance of lateral flow immunochromatography, iELISA and rose Bengal tests for the diagnosis of cattle, sheep, goat and swine brucellosis. PLoS Negl Trop Dis. 2019;13: 1–10. doi: 10.1371/journal.pntd.0007509.
