# Supplementary material for: Latent class evaluation of the performance of serological tests for exposure to Brucella spp. in cattle, sheep, and goats in Tanzania
Source: PLoS Negl Trop Dis. 2021 Aug 24;15(8):e0009630. doi: 10.1371/journal.pntd.0009630 (PMC8384210; doi:10.1371/journal.pntd.0009630)
Supplement: S3 Table — (PDF) [file pntd.0009630.s005.pdf]

S3 Table. Mean sensitivity and specificity estimates for the RBT and cELISA in sheep and goats taken from a literature search of global scientific publications from 1999-2019.

Reference	Methods used for test performance estimation	Location	Species	RBT: Se	RBT: Sp	cELISA: Se	cELISA: Sp	Sample size
Marín *et al*., 1999 [1]	*Se:* Culture positive animals (naturally infected)	Unknown	S	1	1	-	-	115
Minas *et al*., 2005 [2]	*Se:* Culture positive animals (naturally infected)	Greece	S	0.976	0.981	-	-	1017
Burriel *et al*., 2004 [3]	*Se:* Vaccinated animals	Greece	SG	-	-	0.885	0.985	383
Minas *et al*., 2007 [4]	*Se:* Serology positive animals from culture confirmed flocks	Greece	SG	0.866	0.976	-	-	4253
Ramírez-Pfeiffer *et al*., 2008 [5]	*Se:* Animals from area with flock abortions and human cases/PCR or serology positive animals from culture confirmed flocks	Mexico & Canada	G	0.997	0.327	-	-	534
McGiven *et al*., 2009 [6]	*Se:* Culture positive animals (naturally or experimentally infected)/Serology positive animals from culture confirmed flock/Animals from suspected outbreak flock in endemic area	UK & Unknown	SG	-	-	1	0.996	281
Thompson *et al*., 2009 [7]	*Se:* Culture positive animals (naturally or experimentally infected)/Serology positive animals from culture confirmed flock/Animals from suspected outbreak flock in endemic area	UK & Unknown	SG	-	-	1	1	201
Perrett *et al*., 2010 [8]	*Se:* Experimentally infected animals	UK	S<br>G	-<br>-	-<br>-	1<br>1	0.998<br>1	484<br>2023
Weighted mean				0.899	0.919	0.987	0.998	

Se is sensitivity. Specificity was estimated in each study using samples collected from *Brucella* free animals. S is sheep, G is goats and SG is sheep and goats. RBT is the modified Rose Bengal plate test using the 3:1 serum to antigen ratio. cELISA is the Animal and Plant Health Agency, UK, competitive enzyme-linked immunosorbent assay. Weighted mean is the mean value weighted by sample size.

References

1. Marín CM, Moreno E, Moriyón I, Díaz R, Blasco JM. Performance of competitive and indirect enzyme-linked immunosorbent assays, gel immunoprecipitation with native hapten polysaccharide, and standard serological tests in diagnosis of sheep brucellosis. Clin Diagn Lab Immunol. 1999;6: 269–272. doi: 10.1128/CDLI.6.2.269-272.1999.
2. Minas A, Stournara A, Minas M, Papaioannou A, Krikelis V, Tselepidis S. Validation of fluorescence polarization assay (FPA) and comparison with other tests used for diagnosis of *B. melitensis* infection in sheep. Vet Microbiol. 2005;111: 211–221. doi: 10.1016/j.vetmic.2005.10.009.
3. Burriel AR, Christodoulopoulos G, Bisias G, Fthenakis GC. Comparison of fluorescence polarization assay, indirect ELISA and competitive ELISA methods for diagnosis of *Brucella melitensis*-infection in small ruminants. Small Rumin Res. 2004;54: 243–247. doi: 10.1016/j.smallrumres.2003.12.001.
4. Minas A, Stournara A, Minas M, Stack J, Petridou E, Christodoulopoulos G, et al. Validation of a fluorescence polarization assay (FPA) performed in microplates and comparison with other tests used for diagnosing B. melitensis infection in sheep and goats. J Immunol Methods. 2007;320: 94–103. doi: 10.1016/j.jim.2006.12.008.
5. Ramírez-Pfeiffer C, Díaz-Aparicio E, Gomez-Flores R, Rodríguez-Padilla C, Morales-Loredo A, Álvarez-Ojeda G. Use of the *Brucella melitensis* Native Hapten To Diagnose Brucellosis in Goats by a Rapid, Simple, and Specific Fluorescence Polarization Assay. Clin Vaccine Immunol. 2008;15: 911–915. doi: 10.1128/CVI.00046-08.
6. McGiven JA, Thompson IJ, Commander NJ, Stack JA. Time-Resolved Fluorescent Resonance Energy Transfer Assay for Simple and Rapid Detection of Anti- Brucella Antibodies in Ruminant Serum Samples. J Clin Microbiol. 2009;47: 3098–3107. doi: 10.1128/JCM.00919-09.
7. Thompson I, Mcgiven J, Sawyer J, Thirlwall R, Commander N, Stack J. Competitive Electrochemiluminescence Wash and No-Wash Immunoassays for Detection of Serum Antibodies to Smooth *Brucella* Strains. Clin Vaccine Immunol. 2009;16: 765–771. doi: 10.1128/CVI.00006-09.
8. Perrett LL, McGiven JA, Brew SD, Stack JA. Evaluation of competitive ELISA for detection of antibodies to *Brucella* infection in domestic animals. Croat Med J. 2010;51: 314–319. doi: 10.3325/cmj.2010.51.314.
