# Supplementary material for: Latent class evaluation of the performance of serological tests for exposure to Brucella spp. in cattle, sheep, and goats in Tanzania
Source: PLoS Negl Trop Dis. 2021 Aug 24;15(8):e0009630. doi: 10.1371/journal.pntd.0009630 (PMC8384210; doi:10.1371/journal.pntd.0009630)
Supplement: S4 Table — (PDF) [file pntd.0009630.s006.pdf]

S4 Table. Contingency tables comparing RBT and cELISA test results for cattle, sheep, and goat data.

Pastoral: Cattle

	cELISA negative	cELISA positive
**RBT negative**	1922	53
**RBT positive**	22	67

Non-pastoral: Cattle

	cELISA negative	cELISA positive
**RBT negative**	1379	12
**RBT positive**	3	9

Pastoral: Sheep

	cELISA negative	cELISA positive
**RBT negative**	1666	37
**RBT positive**	14	22

Non-pastoral: Sheep

	cELISA negative	cELISA positive
**RBT negative**	752	2
**RBT positive**	12	3

Pastoral: Goat

	cELISA negative	cELISA positive
**RBT negative**	1771	40
**RBT positive**	25	56

Non-pastoral: Goat

	cELISA negative	cELISA positive
**RBT negative**	1246	3
**RBT positive**	9	16

Cattle, sheep, and goat data are from the combined BacZoo study conducted 2013 to 2015 in Arusha and Kilimanjaro Regions, and the SEEDZ study conducted in 2016 in Arusha and Manyara Regions of Tanzania. RBT is Rose Bengal plate test. For sheep and goat models, RBT refers to the modified RBT 3:1 serum to antigen ratio. cELISA is the Animal and Plant Health Agency, UK, competitive enzyme-linked immunosorbent assay. The left-side column shows diagnostic test data in the pastoral subpopulation for each livestock species, and the right-side column shows diagnostic test data in the non-pastoral subpopulation for each livestock species.
