# Supplementary material for: Latent class evaluation of the performance of serological tests for exposure to Brucella spp. in cattle, sheep, and goats in Tanzania
Source: PLoS Negl Trop Dis. 2021 Aug 24;15(8):e0009630. doi: 10.1371/journal.pntd.0009630 (PMC8384210; doi:10.1371/journal.pntd.0009630)
Supplement: S3 Fig — (PDF) [file pntd.0009630.s009.pdf]

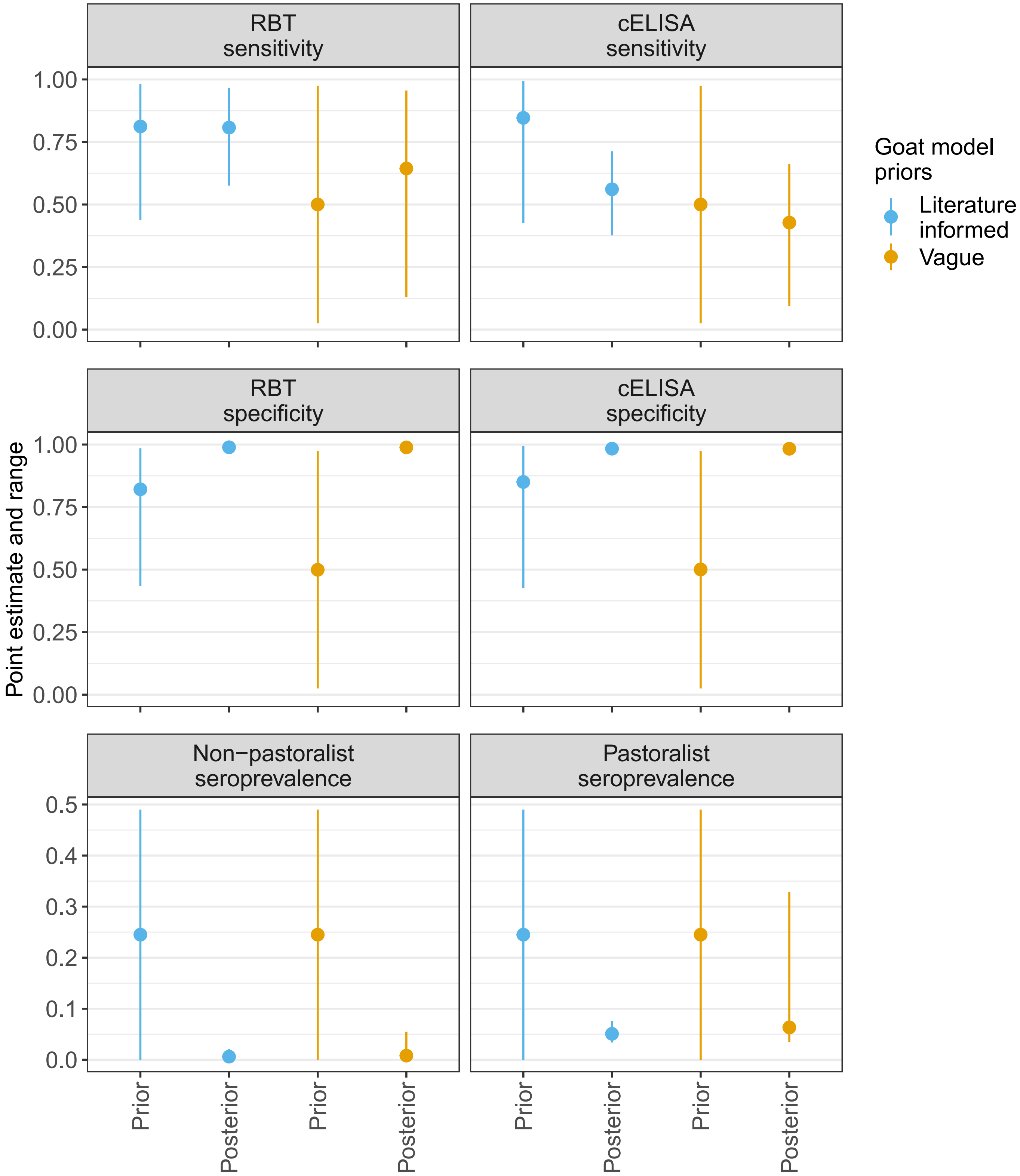


**S3 Fig. Sensitivity, specificity and seroprevalence point estimates and ranges for literature informed and vague uniform goat model priors and posteriors.** Goat data are from the combined BacZoo study conducted 2013 to 2015 in Arusha and Kilimanjaro Regions, and the SEEDZ study conducted in 2016 in Arusha and Manyara Regions of Tanzania. Point estimate refers to beta distribution mode for priors, and the median estimate for posteriors. Range refers to the beta distribution range for priors, and 95% Bayesian credibility interval for posteriors. RBT is Rose Bengal plate test. For sheep and goat models, RBT refers to the modified RBT 3:1 serum to antigen ratio. cELISA is the Animal and Plant Health Agency, UK, competitive enzyme-linked immunosorbent assay. Literature informed estimates were generated using a literature search of scientific publications from 1999-2019. Goat estimates were calculated from studies from varied geographic locations. Vague priors were specified as dbeta(1,1).
