# Supplementary material for: Latent class evaluation of the performance of serological tests for exposure to Brucella spp. in cattle, sheep, and goats in Tanzania
Source: PLoS Negl Trop Dis. 2021 Aug 24;15(8):e0009630. doi: 10.1371/journal.pntd.0009630 (PMC8384210; doi:10.1371/journal.pntd.0009630)
Supplement: S5 Fig — (PDF) [file pntd.0009630.s011.pdf]

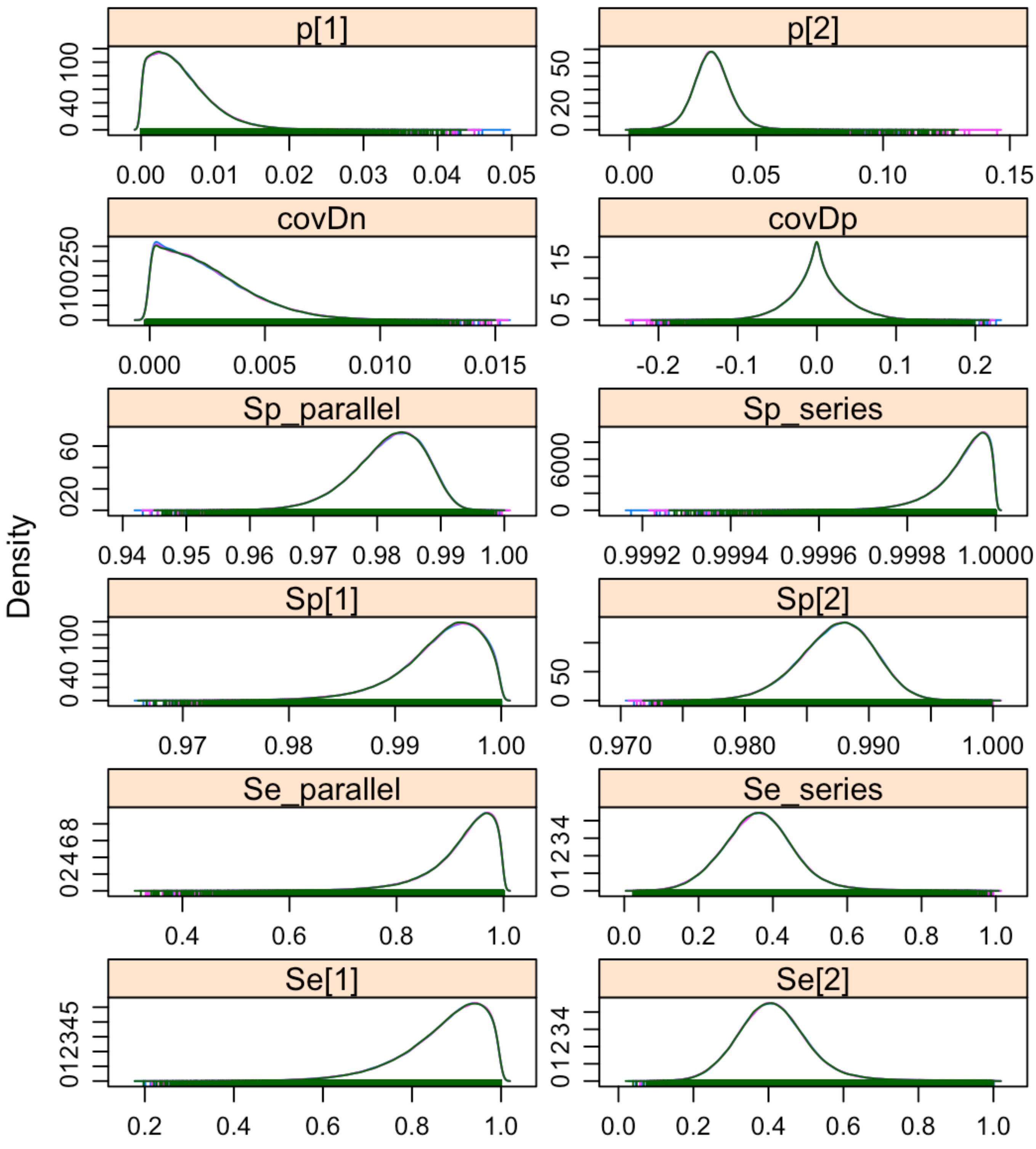

S5 Fig. Density plots of sheep model parameters. Sheep data are from the combined BacZoo study conducted 2013 to 2015 in Arusha and Kilimanjaro Regions, and the SEEDZ study conducted in 2016 in Arusha and Manyara Regions of Tanzania. p[1] denotes seroprevalence in the non-pastoralist subpopulation. p[2] denotes seroprevalence in the pastoralist subpopulation. Se is sensitivity. Sp is specificity. [1] denotes the Rose Bengal plate test using the modified RBT 3:1 serum to antigen ratio. [2] denotes the Animal and Plant Health Agency, UK, competitive enzyme-linked immunosorbent assay. series is a series diagnostic testing approach. parallel is a parallel diagnostic testing approach. covDn is covariance when animal is disease negative. covDp is covariance when animal is disease positive.
